# Microphthalmia with Linear Skin Defects (MLS) associated with Autism Spectrum Disorder (ASD) in a patient with Familial 12.9Mb Terminal Xp deletion

**DOI:** 10.1186/1471-2431-14-220

**Published:** 2014-09-02

**Authors:** Lucia Margari, Annalisa Colonna, Francesco Craig, Mattia Gentile, Giustina Giannella, Anna Linda Lamanna, Anna Rosi Legrottaglie

**Affiliations:** 1 Child Neuropsychiatry Unit, Department of General Medicine, Neuroscience and Sensory Organs of the “Aldo Moro” University of Bari, Bari, Italy; 2 Department of Medical Genetics, Hospital Di Venere, ASL BARI, Bari, Italy

**Keywords:** MLS syndrome, Autism spectrum disorder, Xp deletion, X-inactivation, Phenotypic variability

## Abstract

**Background:**

Microphthalmia with linear skin defects (MLS) syndrome is a rare X-linked dominant male-lethal developmental disorder characterized by unilateral or bilateral microphthalmia and linear skin defects of the face and neck. Additional features affecting the eyes, heart, brain or genitourinary system can occur, corroborating the intra- and interfamilial phenotypic variability. The majority of patients display monosomy of the Xp22.2 region, where the holocytochrome c-type synthase (HCCS) gene is located.

**Case presentation:**

We describe a 15-year-old-female affected by MLS syndrome and autism spectrum disorder (ASD). ASD has not previously been reported as a component of MLS. Our patient shows a large deletion of 12.9 Mb, involving Xp22.32-p22.2, which encompasses both the HCCS gene and autism X-linked genes.

**Conclusion:**

Thus, patients with a large deletion at Xp22 might display MLS with ASD, due to the deletion of contiguous genes, although the highly variable phenotype of these patients could be influenced by several genetic mechanisms, including different tissue-specific X-inactivation and somatic mosaicism.

## Background

First described in 1988, the microphthalmia with linear skin defects (MLS) syndrome (OMIM 309801) is also known as MIDAS (Microphthalmia, Dermal Aplasia, Sclerocornea) syndrome [1], but the MLS acronym is more appropriate since dermal aplasia has never been described in histologic samples [2]. This rare X-linked dominant disease with male lethality *in utero* belongs to neurocutaneous development disorders, and is characterized by unilateral or bilateral microphthalmia as well as linear skin defects, along Blaschko lines, limited to the face and neck. Most patients with MLS display the classical phenotypic features; however, a high intra- and interfamilial clinical variability exists, and additional features affecting the eyes, heart, brain and genitourinary system may be present [3].

Conventional and molecular cytogenetic studies of the majority of patients with MLS syndrome have revealed segmental monosomy of the Xp22 chromosomal region. In particular, the MLS critical region has been identified at 610 Kb in Xp22.2, and includes the *holocytochrome c-type synthase* (*HCCS*) gene*,* encoding the mitochondrial holocytochrome *c*-type synthase, that is the gene whose mutation is known to cause MLS syndrome [4,5]. Wimplinger et al. [5] detected that the final product of HCSS activity is cytochrome c, which plays a key role in apoptosis. Therefore *HCCS* mutations impair apoptosis, leading to an abnormal eye and brain embryonic development. It has been also hypothesized that a disturbance of both the oxidative phosphorylation and the balance between apoptosis and necrosis, as well as tissue specific X-inactivation patterns, may contribute to the variable phenotype [5].

Here we report the first case of MLS syndrome with associated ASD features. Our patient shows a large deletion involving Xpter-p22.2. The deletion encompasses both the *HCCS* gene and several autism X-linked genes, suggesting that large Xp deletions might be associated with both MLS and ASD, probably due to the deletion of contiguous genes. However, several genetic mechanisms can influence the highly variable phenotype of this exceedingly rare disorder.

## Case presentation

The proband, a 15-year-old female, was born to a 28 year-old father and a 27 year-old mother. Her father and sister are phenotypically normal. The mother presented a relatively short stature (cm 160), and corneal degeneration, diagnosed at birth, for which she underwent enucleation, and left eye prosthesis. Family history was remarkable for not otherwise specified anxiety disorders, and epilepsy in the maternal line.

An intrauterine growth restriction was revealed at the 22^th^ gestational week. She was born at 38 weeks gestation via emergency cesarean section, due to fetal distress. Birth weight was 2.530 gr (10^th^ centile), length 47 cm (10-50^th^ centile) and head circumference 33 cm (10-50^th^ centile). The Apgar scores were 7 and 8. Immediately after birth, several life-threatening respiratory distress events occured, requiring patient intubation. Slight asymmetry of the cerebral ventricles with hyperechogenicity of the parenchyma on the superior-lateral horns of the lateral ventricles was detected at ultrasound examination of the brain.

The newborn female showed linear and erythematous skin lesions on the cheek, extending to the nose and chin. Ophthalmologic evaluation revealed leukoma cornea and microphthalmia of the right eye, and sclerocornea and anophthalmia of the left. The clinical diagnosis of the MLS syndrome was made.

Motor development was slightly delayed: she walked independently at 2 years of age, as commonly observed in blind children, instead speech development was normal until 18–24 months of age (she was able to speak simple sentences), after that she started to show echolalia and verbal stereotypy. At the age of 30 months, a further deterioration of language and cognitive abilities occurred, with loss of previously acquired skills: expressive language was limited to two-three single words, and unintelligible verbalizations. She gradually developed seriously impaired communication, socialization and cognition skills, as well as sleep disturbances (difficulty falling asleep).

**Figure 1:**
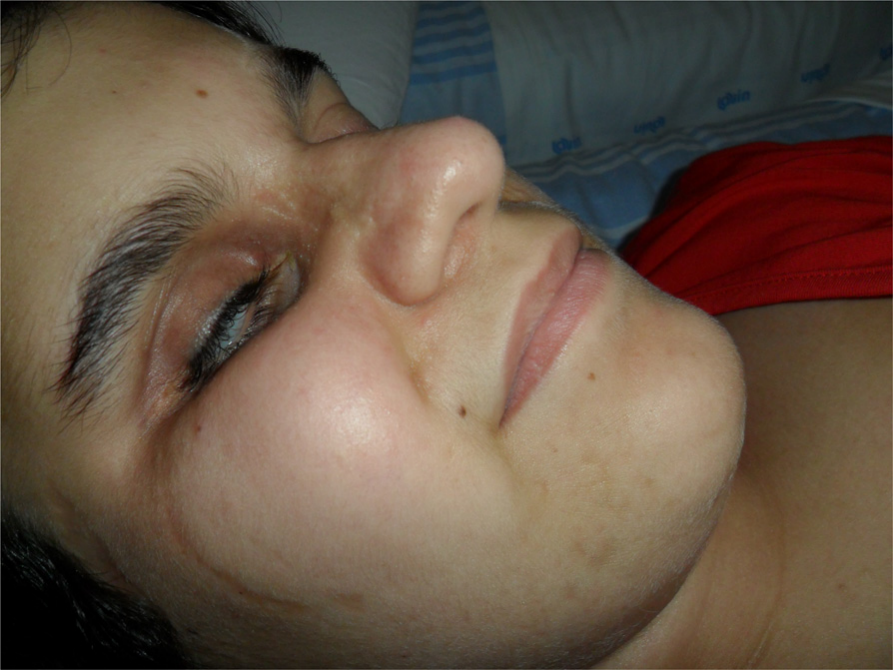
**Dysmorphic features in the patient.** Physical examination revealed areas of scar-like aplasia cutis following the Blaschko lines along the cheek, nose and chin.

The patient was admitted to our department at the age of 15 years, due to progressive social avoidance and self-harm behaviors developing during the last year. Physical examination revealed skeletal abnormalities such as micromelia, lower limb asymmetry, left scoliosis and *pes cavus* bilaterally. Our patient had evident dysmorphic features, including microcephaly (head circumference < 3^rd^ centile), flattened occiput, hypotelorism, low setting ears, micrognathia, height and weight below the third percentile, and a broad based gait. In addition, she showed areas of scar-like aplasia cutis following the Blaschko lines along the cheek, nose and chin (Figure 1). Neurologic examination showed diffuse hypotonia. An ophthalmologic evaluation revealed right eye microphthalmia with opaque cornea and left eye anophthalmia (with a prosthesis), with total blindness in the left eye, and perception of light in the right one.

Cardiac and otorinolaringoiatric examinations were normal, as well as auditory brainstem responses and asleep electroencephalography. Brain magnetic resonance imaging showed a slight hyperintensity in the peritrigonal white matter bilaterally, probably due to neonatal distress. Routine blood tests, plasma amino acids, urine organic acids and lactate were unremarkable.

Psychiatric and behavioral assessments, conducted by direct observation of the patient’s spontaneous behavior, revealed social withdrawal. Indeed, she preferred to be alone, she did not turn around if her name was called, and she did not respond to instructions and appeared distant when attempts were made to interact with her. She showed limited emotional non-verbal contact and lacked social skills. Communication was limited to two-three single words and vocalizations such as vowel sounds or unintelligible verbalizations and failure to compensate through gesture. She also showed stereotypic and repetitive motor mannerisms, many restricted, ritualistic and “sameness” behaviors (e.g., listening to the same music all the time), that were not due to visual impairment. Moreover, she displayed self-harm tendencies and restricted personal autonomy. Autistic features identified in our patient were not due to visual impairment and were confirmed by standardized rating scales including the Childhood Autistic Rating Scale (CARS), Autism Diagnostic Interview-Revised (ADI-R), Vineland Adaptive Behavior Scale. Indeed, on the CARS she scored 44 points (cut-off score 30), reflecting severe autism. The diagnosis was confirmed by the ADI-R. The Vineland Adaptive Behavior Scale showed severe deficits in adaptive behaviors. Her cognitive abilities were not assessed using the standardized intelligence test because of the severe blindness. However, empirical judgment suggested moderate intellectual disability.

A diagnosis of the MLS syndrome with Autism Spectrum Disorder (ASD) and moderate intellectual disability was made, according to the Diagnostic and Statistical Manual of Mental Disorders 5th Edition (DSM-V) criteria. Written informed consent was obtained from the parent of the patient.

### Genetic investigation

**Figure 2:**
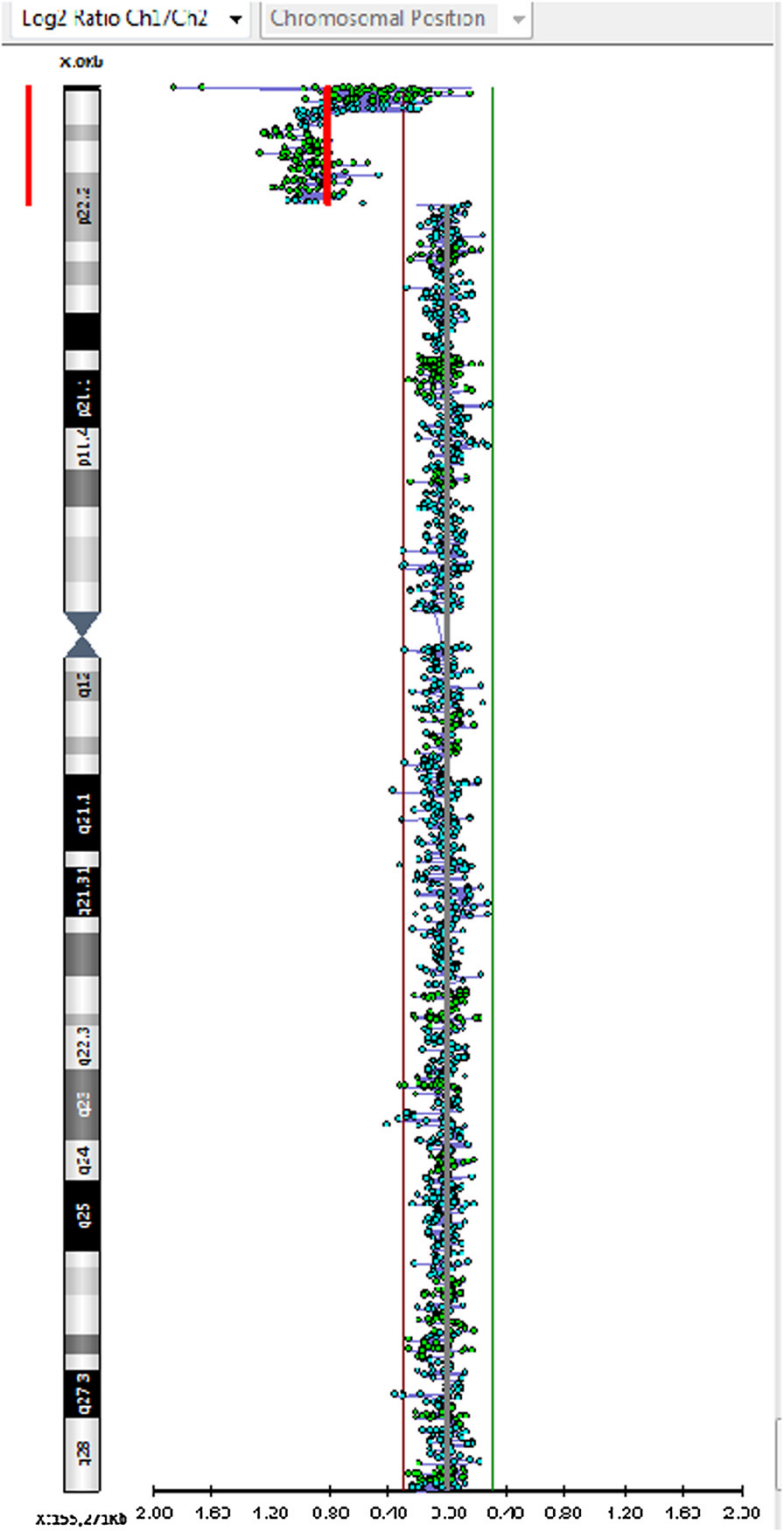
Array CGH analysis with a genomic resolution of 75 Kb showing a terminal microdeletion of about 12,9 Mb into Xp22.2-pter region.

Genomic DNA was extracted from peripheral blood samples. DNA concentration was measured by fluorimeter (Amersham, Piscataway, NJ) using the Hoechst reagent and adjusted to 400 ng/ml.Array-CGH analysis was performed using the Cytochip oligo ISCA 4x44K (Techno Genetics Srl, Italy). This array is composed of 60-mer oligonucleotides spaced at about 75 Kb density across the genome. Labeling and hybridization were carried out following the manufacturer’s protocol. Slides were scanned on the InnoScan 710 (Innopsys), and analyzed using the BlueFuse Multi v3.1 (Bluegnome). The analysis revealed a terminal deletion of the short arm of chromosome X, with a loss in copy number at the Xp22.33p22.2 region [chrX:702–12,949,422 (hg19), size 12,948,721 bp] (Figure 2).

Result of array CGH analysis were confirmed by FISH. BAC probes RP11-509B6, RP11-109P4, and RP11-102 M2 (Bluegnome), mapping in the deleted region, produced hybridization signals only on the normal chromosome X. The same deletion was revealed in the patient’s mother, and confirmed by FISH analysis which also excluded chromosomal mosaicism on interphase nuclei.

Therefore the final karyotype of the patient was designed as (ISCN 2013): 46,XX.arr Xp22.33p22.2(702–12,949,422)x1 mat.

X inactivation state was investigated using HUMARA X-chromosome assay [6]. X-chromosome inactivation was skewed, with preferential inactivation of the X chromosome carrying the terminal deletion (ratio of 10% to 90% in the proband; 15% to 85% in the mother).

## Conclusion

In our patient, aCGH analysis has revealed a large terminal deletion of 12,9 Mb (Xp22.2 → Xp22.33), with proximal breakpoint at 12,949,422. The deletion was confirmed by FISH; an identical imbalance was present in the mother. The deletion involves 52 OMIM genes, and is associated with several diseases.

In male patients, Xp deletions result in a contiguous gene syndrome [7,8], and become lethal *in utero* when extend beyond 9.7 Mb from Xpter [9], or include some regions, i.e. the 610 Kb minimal critical region for MLS, with loss of the *HCSS* gene [10]. Females with terminal Xp deletions are characterized by a milder phenotype with highly variable clinical manifestations, depending on the size of the deletion as well as from other factors, such as tissue specific X-inactivation patterns and/or chromosomal mosaicism [4].

Considering our patient phenotype, we can attribute the proportionate short stature (< 3rd centile) and the skeletal abnormalities to the loss of the *SHOX* gene, which is located in the telomeric portion of the pseudoautosomal region 1 (PAR1). The gene is dose-dependent, and escape X inactivation whereby its loss may cause Léri-Weill syndrome or non syndromic short stature [11].

In addition, in our case the loss of the Xp22.2 region includes the genes *MID1*, *HCCS*, and *ARHGAP6*, and is associated with MLS [1-3]. Our patient shows MLS major criteria (right eye microphthalmia and left anophthalmia, areas of scar-like aplasia cutis along the cheek, nose and chin), and several minor criteria, namely right cornea leukoma, left sclerocornea, developmental delay, intellectual disability and dysmorphic features. The mother, with the same deletion, shows a milder phenotype with left eye corneal degeneration as the only sign attributable to MLS. Otherwise this is not surprising, being well known in literature the very high intra- and interfamilial variability of MLS [3,4,12]. Several mechanism has been proposed. In some family chromosomal mosaicism can account for the clinical variability [4]. However in our case such mechanism has been excluded by FISH analysis, and X-inactivation in blood cells does not explain the variable phenotype observed in the family. Therefore, according to literature, different tissue specific X-inactivation patterns seem the more plausible reason for the mild vs severe mother/daughter phenotype [13,14].

Finally, a further, more peculiar, part of our patient phenotype regards autistic behavioral features. Our patient fulfilled DSM-V criteria for ASD on persistent deficits in social communication and social interaction across contexts and repetitive patterns of behavior, interests, or activities.

While developmental delay and intellectual disability have been reported in a few patients, to the best of our knowledge ASD has never previously been recognized in MLS patients [3,15]. Differently large Xp deletions seem associated with learning impairment and autistic tendencies, even if the published cases are very rare, and a detailed and thorough developmental assessment of ASD has never been reported [10].

In particular Chocholska et al. [13] described a family with an interstitial 7.7 Mb Xp22.2-22.3 deletion: the son (III/2) showed autism and developmental delay. Hobson et al. [10] reported a 20-year-old female with a terminal Xp deletion very similar to our case (proximal breakpoint at 12,024,000 bp) with short stature, MLS, moderate learning difficulty associated with autistic traits (obsessive behaviors, routines, lack of empathy). Furthermore autism, developmental delay, and dysmorphic features were present in a 10-year-old patient with a de novo interstitial Xp22 deletion extending from 5,005,810 to 10,556,218 bp [16].

Therefore we can affirm that Xp22 region can play a pivotal role in the pathogenesis of autism, based on the phenotype of the rare patients with large Xp22 deletion as well as on the presence of several genes potentially associated with a behavioral phenotype that fits within the autism spectrum disorder, i.e. *NLGN4X, VCX* genes [10,13,16-18]. NLGN proteins are postsynaptic adhesion proteins, expressed in brain, essential for synapse organization and function [19]. Mutations/haploinsufficiency of *NLGN4X* seem involved in the pathogenesis of ASD [16,20], but it's not clear if such role regards a limited fraction (<1%) of ASD patients or *NLGN4X* variants could account for a more relevant role in ASD [21]. *VCX* genes role in cognitive impairment has been demonstrated; in addition these genes might contribute to the onset of neuropsychiatric illness, i.e. attention deficit hyperactivity disorder [16]. Considering all these evidences, we can assume that the presence of ASD seems related to the loss of the Xp22.32p22.31 region containing *NLGN4X/VCX* genes, and not to the more proximal MLS critical region. However further cases are needed to draw more definitive issues.

In conclusion, the description of our case lends further support to the notion that females with large deletions of Xp or a 45,X constitution may be at increased risk of autism as the result of deletions of autism-linked genes [17], indicating the need to carry out a detailed neuropsychological assessment in patients with MLS to check for ASD, especially when MLS critical region haploinsufficiency occurs in the context of a large Xp deletion. In these cases a multi-disciplinary approach to the management of children with the MLS syndrome is highly desirable. Moreover, genotype/phenotype correlation studies are needed to better characterize the contribution of the individual genes mapping in Xp22 to the autistic spectrum disorders.

### Consent

Written informed consent was obtained from the parent of patient for publication of this Case report and any accompanying images. This manuscript represents a descriptive, retrospective case report without any intervention, and, therefore, it did not require review or approval by the Ethical Committee “Azienda Ospedaliero-Universitaria Consorziale Policlinico” at our hospital. A copy of the written consent is available for review by the Editor of this journal.

## Abbreviations

MLS: Microphthalmia with linear skin defects syndrome; HCCS: Holocytochrome c-type synthase; ASD: Autism spectrum disorder; MIDAS: Microphthalmia, dermal aplasia, sclerocornea; aCGH: Array comparative genomic hybridization analysis; CARS: Childhood autistic rating scale; ADI-R: Autism diagnostic interview-revised; DSM-V: Diagnostic and statistical manual of mental disorders 5th edition.

## Competing interests

We confirm that all co-authors have seen and approved the final version of the paper and accept responsibility for the data presented, and that there is no financial or others conflict of interest that may be related to the authors.

## Authors’ contributions

LM: Conception, design and interpretation, revising it critically for important intellectual content, final approval of the article. AC: Data collection, literature research, and wrote the first draft of the manuscript. FC: Data collection, drafting the article, neuropsychological assessment and English writing style. MG: analysis (FISH, array CGH), interpretation and revision of the genetic data. GG: was the neurology resident who performed the in-patient consult, critical revision of the article. ALL: Provision of materials and patient, drafting the article. ARL: Data collection and literature research. All authors read and approved the final manuscript.

## Pre-publication history

The pre-publication history for this paper can be accessed here:

http://www.biomedcentral.com/1471-2431/14/220/prepub
